# Recent genome resequencing paraded COBRA-*Like* gene family roles in abiotic stress and wood formation in Poplar

**DOI:** 10.3389/fpls.2023.1242836

**Published:** 2023-09-15

**Authors:** Muhammad Sajjad, Adeel Ahmad, Muhammad Waheed Riaz, Quaid Hussain, Muhammad Yasir, Meng‐Zhu Lu

**Affiliations:** ^1^ State Key Laboratory of Subtropical Silviculture, College of Forestry and Biotechnology, Zhejiang A & F University, Hangzhou, China; ^2^ State Key Laboratory of Cotton Biology, Institute of Cotton Research, Chinese Academy of Agricultural Sciences, Anyang, Henan, China; ^3^ Zhejiang Provincial Key Laboratory of Resource Protection and Innovation of Traditional Chinese Medicine, Zhejiang A&F University, Hangzhou, China; ^4^ Shenzhen Key Laboratory of Marine Bioresource and Eco-Environmental Science, College of Life Sciences and Oceanography, Shenzhen University, Shenzhen, China; ^5^ The Key Laboratory for Quality Improvement of Agricultural Products of Zhejiang Province, College of Advanced Agricultural Sciences, Zhejiang A&F University, Hangzhou, China

**Keywords:** cell wall, COBRA-*Like*, genome-wide, abiotic stress, tension wood, RNA-Seq

## Abstract

A cell wall determines the mechanical properties of a cell, serves as a barrier against plant stresses, and allows cell division and growth processes. The COBRA-*Like* (COBL) gene family encodes a putative glycosylphosphatidylinositol (GPI)-anchored protein that controls cellulose deposition and cell progression in plants by contributing to the microfibril orientation of a cell wall. Despite being studied in different plant species, there is a dearth of the comprehensive global analysis of COBL genes in poplar. Poplar is employed as a model woody plant to study abiotic stresses and biomass production in tree research. Improved genome resequencing has enabled the comprehensive exploration of the evolution and functional capacities of PtrCOBLs (Poplar COBRA-Like genes) in poplar. Phylogeny analysis has discerned and classified PtrCOBLs into two groups resembling the *Arabidopsis* COBL family, and group I genes possess longer proteins but have fewer exons than group II. Analysis of gene structure and motifs revealed PtrCOBLs maintained a rather stable motif and exon–intron pattern across members of the same group. Synteny and collinearity analyses exhibited that the evolution of the COBL gene family was heavily influenced by gene duplication events. PtrCOBL genes have undergone both segmental duplication and tandem duplication, followed by purifying selection. Promotor analysis flaunted various phytohormone-, growth- and stress-related cis-elements (e.g., MYB, ABA, MeJA, SA, AuxR, and ATBP1). Likewise, 29 Ptr-miRNAs of 20 families were found targeting 11 PtrCOBL genes. PtrCOBLs were found localized at the plasma membrane and extracellular matrix, while gene ontology analysis showed their involvement in plant development, plant growth, stress response, cellulose biosynthesis, and cell wall biogenesis. RNA-seq datasets depicted the bulk of PtrCOBL genes expression being found in plant stem tissues and leaves, rendering mechanical strength and rejoinders to environmental cues. PtrCOBL2, 3, 10, and 11 manifested the highest expression in vasculature and abiotic stress, and resemblant expression trends were upheld by qRT-PCR. Co-expression network analysis identified PtrCOBL2 and PtrCOBL3 as hub genes across all abiotic stresses and wood developing tissues. The current study reports regulating roles of PtrCOBLs in xylem differentiating tissues, tension wood formation, and abiotic stress latency that lay the groundwork for future functional studies of the PtrCOBL genes in poplar breeding.

## 1 Introduction

Forests are greatly important for this world, particularly in an era of huge climate change, which is bringing about drastic changes around the globe, e.g., temperature hikes, droughts, and floods (Rudel et al., 2020). Secondly, the growing global population is causing a surge in demand for wood and tree-based products. To address these issues, we need to understand the regulatory mechanisms in tree growth, development, and stress tolerance so that we can produce genetically better trees with improved wood properties and stress resilience (De Frenne et al., 2021).

The cell wall is a flagship component of plant cells that provides mechanical strength and plasticity for expansion to the cells. As a result of cell division and expansion, plant cells differentiate into numerous ultimate shapes and forms throughout their life span (Coleman et al., 2021). The cell wall determines the shape of a plant cell, which is achieved through a combination of directed cell division and cell expansion. The form of a plant cell is essential to its function, and its shape can be maneuvered in response to biotic and abiotic signals (Brady et al., 2007; Yoshida et al., 2021). The cell wall of a plant is a dynamic, fibrillar nexus. After a plant cell has reached its ultimate shape and the primary cell wall has been established, the secondary cell wall forms during the development of woody tissues (Xie et al., 2018; Zhang et al., 2018). Cellulose is a microfibrillar polymer that is found in high concentrations in plant cell walls. Cellulose synthase (CESA) proteins, which dwell in the plasma membrane inside cellulose synthase complexes (CSCs), are responsible for the formation of these microfibrils (McFarlane et al., 2014; Gritsch et al., 2015). COBLs are found to be associated with cellulose crystallizations and decisively co-expressed with cellulose synthase complex (CSC) (Sangi et al., 2021; Li Z. et al., 2022). Cellulose synthases (CESAs), KORRIGAN (KOR), NAC transcription factor, chitinase-like genes (CTLs), fasciclin-like arabinogalactan genes (FLAs), and MYB transcription factors are only a few of the genes that have been shown to be crucial for cellulose biosynthesis during the past several decades (Liu et al., 2013; Yoshida et al., 2021). Despite these significant advances, the molecular process of cellulose production and deposition is still poorly understood. Veritably, it is still unclear how plants naturally organize the molecules in their cellulose framework. Little is known regarding the proteins and the mechanisms that regulate cellulose crystallinity in plants (Somerville, 2006; Niu et al., 2015); this is where COBL proteins come into play. But there has not been enough research done on COBRA-*Like* genes in tree species like poplar to draw any firm conclusions. COBRA belong to a plant-specific multigene (COB-like) family and encode glycosylphosphatidylinositol (GPI)-anchored proteins embodying the hydrophilic region at the C-terminal, typically having an ω-attachment site for GPI modification accompanying an N-terminal secretion signal (Liu et al., 2013; Yang et al., 2021). COB protein is localized in vesicles, in the Golgi apparatus, and lastly at the cell surface, conforming to a classic GPI secretion pathway. The microtubule arrangement determines where the COB protein is distributed (Sato et al., 2010; Zhang et al., 2016).

The COBL family members have been identified to intercede in several developmental and physiological processes, including stem strength, pollen tube growth, pathogen resistance, and root-hair growth (Ko et al., 2006; Li et al., 2013; Yang et al., 2021; Li et al., 2022). Many mutants with aberrant cell expansion and cellulose deposition have been investigated to learn about the molecular processes that govern the direction and process of cell expansion during plant development (Ko et al., 2006; Hochholdinger et al., 2008). In *Arabidopsis*, cobra-mutants like *cob-6* are unable to produce cellulose, which leads to defective growth (Sato-Izawa et al., 2020). A decrease in the frequency of mis-regulated genes and a suppression of symptoms are seen in *cob-6* mutants with mutations in MEDIATOR16 (MED16) (Sorek et al., 2015). This finding suggests a role for MED16 in the transcriptional response to a breach in the cell wall (Sorek et al., 2015). *Arabidopsis* COBL2 controls seed mucilage ray shaping, adhesion, and crystalline cellulose deposition into the cell walls. In contrast, *cobl2* triggers impaired cell wall assembly and morphology (Ben-Tov et al., 2018). COBL10 likely regulates pollen tube growth by the deposition of cellulose microfibrils and apical pectin (Li et al., 2013). *cobl4* mutants exhibit phenotypes with impaired secondary cell wall (SCW) formation (Liu et al., 2013). *AtCOBL9* is mandatory for root hair growth whereas *AtCOBL4* is found to be vital for SCW cellulose generation in *Arabidopsis* (Dai et al., 2009). In rice, *BC1* (*Brittle Culm1*) participation is required for cellulose biogenesis and crystallization. While *BC1* mutation causes enfeebled stem strength. In maize, *Brittle Stalk2* (BK2) mutation begets defective mechanical strength (Li et al., 2003; Julius et al., 2021). The epidermal cells of a tomato mutant with the *SlCOBRA-like* gene silenced lacked cuticles, with aberrant shapes and an erratic size distribution. Plants with loss-of-function COBL genes, such as *BRITTLE CULM1* in sorghum (*SbBC1*) and *TmBr1* in diploid wheat, induce the brittle phenotype (Li et al., 2018). One study also reported that plant cells interpret the *COBRA* null mutation’s effects on cell elongation as a hint of an impending pathogen assault and adapt their growth accordingly (Ko et al., 2006). The *cob-5* mutant has aberrant cell development across the whole plant body and agglomerates compounds that are released in reaction to stress, e.g., anthocyanins and callose. Further investigation revealed that *cob-5* plants increased JA concentration thus dramatically increasing the regulation of their cellular defense mechanisms (Ko et al., 2006; Gao et al., 2013). These findings provide credence to the hypothesis that modifications in cell wall integrity are closely linked to the control of plant defense mechanisms (Yoshida et al., 2021).

Poplar is a model plant in tree research and a valuable forest resource to study wood formation mechanisms and stress response (Liu et al., 2015). The COBL gene family modulates wood developing tissues (secondary xylem, phloem, xylem vessel, and fiber differentiation) involved in stem girth and stress response (Zaheer et al., 2021). In *Populus*, one study (Ye et al., 2009) found 18 COBRA genes, but an inclusive and systematically analyzed study is lacking. Recent and advanced *Populus* genome sequencing can greatly improve our understanding about the functions, evolution, gene distribution, and expansion of the COBL gene family in poplar (Tuskan et al., 2006; Zhang et al., 2022). Here, we present a comprehensive study on the COBL gene family in poplar. In the current study, chromosomal collinearity with different plant species and expression patterns under abiotic stress and in different tissues was evaluated. Furthermore, miRNA regulation and gene ontology (cellular, molecular, and biological processes) of COBL genes were investigated. PtrCOBL genes were identified regulating tension-wood formation and co-expression links among these genes were explored. The current study will better equip the researchers to use the key PtrCOBL genes in cutting-edge genome editing procedures that will assist their functional assessment and may result in superior genotypes with improved wood quality and stress tolerance capacity.

## 2 Materials and methods

### 2.1 Evolutionary relationships and search for COBLs

Poplar and *Arabidopsis* COBRA-*Like* family protein sequences were replevied from the Phytozome13 and TAIR online databases, respectively (https://phytozome.jgi.doe.gov/pz/portal.html and https://www.arabidopsis.org/ (Accessed on 01 Sep 2022)) (Xu et al., 2017). After retrieving the COBL domain (PF04833) from Pfam (http://pfam.xfam.org/ (Retrieved on 01 Sep 2022)), we used it as a query to search other plant species protein databases using HMMER 3.0 to identify the genes that contained this domain (Potter et al., 2018). The predicted genes were verified using the SMART database (http://smart.embl-heidelberg.de/) and the NCBI CD search tool (https://www.ncbi.nlm.nih.gov/Structure/cdd/wrpsb.cgi) to look for conserved domains and motifs. To determine the COBL proteins’ chemical and physical properties, the ExPASy database (http://web.expasy.org/protparam/) was employed. For protein sequence alignment, ClustalW (Larkin et al., 2007) and DNAMAN (http://www.lynnon.com/) software were used. For the phylogenetic tree construction, muscle alignment was utilized coupled with MEGA-X (http://www.megasoftware.net/mega-x/) and its neighbor-joining (NJ) approach. The numbers at the tree branches indicate the percentage (%) of 1,000 bootstrap values (Kumar et al., 2018).

### 2.2 Gene structure prediction, domain, and motif analyses

The genomic sequences of poplar were obtained from the Phytozome13 database. To examine the COBL family gene-structure (exon, intron, and UTR), we utilized the web-based GSDS program (http://gsds.cbi.pku.edu.cn/). The conserved motifs were analyzed with default settings using the online freeware MEME (http://meme-suite.org/tools/meme), and the motifs were visualized using the TBtools program (Chen et al., 2020). The Conserved Domains Database (CDD) (https://www.ncbi.nlm.nih.gov/Structure/bwrpsb/bwrpsb.cgi) was used to identify COBLs conserved domains. The TBtools program was used to draw the domain structure with default parameters (Chen et al., 2020).

### 2.3 PtrCOBLs mapping on chromosomes and gene duplication

The Phytozome and PopGenIE (http://popgenie.org/chromosome-diagram) databases were mined for chromosomal location data of PtrCOBLs (Leng et al., 2021). Every single gene was mapped on the representing chromosome based on its position report. Whole genome duplication (WGD) occurrences were analyzed using MCScanX. The synteny and collinearity analyses were visualized between poplar and five other plant species (*Arabidopsis*, *eucalyptus*, cotton, *T. cacao*, and *V. vinifera*) using TBtools with default settings (Chen et al., 2020; Yang et al., 2021).

### 2.4 Analysis of cis-regulatory elements and sub-cellular localization

To carry out promotor analysis, 2KB upstream sequences (from the start codon) of COBL genes were submitted to online freeware, Plant-CARE (Lescot et al., 2002) (http://bioinformatics.psb.ugent.be/webtools/plantcare/html/). The subcellular localization of COBL genes was predicted using the CELLO web server (http://cello.life.nctu.edu.tw/).

### 2.5 Putative miRNA targeting COBLs and analysis of their functions

The psRNATarget website was used to estimate miRNA target sites. The CDS sequences of all the COBL genes were submitted to the website (https://www.zhaolab.org/psRNATarget/) under default parameters. Moreover, these miRNAs and target sites were verified with another tool (Raza et al., 2022). The interactive network of miRNAs with their sites was developed utilizing Cytoscape software (v3.9). All of the PtrCOBL protein sequences were presented to the eggNOG v4.0 for review of gene ontology (GO) and KEGG (Kyoto encyclopedia of genes and genomics) annotations (Bano et al., 2021; Raza et al., 2022).

### 2.6 Gene co-expression network analysis

The PtrCOBL gene expression was measured in terms of fragments per kilobase of exon per million mapped (FPKM). Differentially expressed COBL genes were studied in vegetative organs, vasculature (wood-making tissues), and in response to abiotic stresses using transcriptome data (NCBI (ID: GSE153793) ENA (ID: ERP016242)) from NCBI (National Center for Bio-technology Information) (https://www.ncbi.nlm.nih.gov/) (Raza et al., 2022). The heat map was produced using TBtool software and the FPKM data were used to conduct co-expression network analysis. Cytoscape was used to generate a co-expression network out of the collected data (Shannon et al., 2003; Chen et al., 2020).

### 2.7 Bioinformatic analysis of potential distinct features of COBLs

The PlantRegMap online database (http://plantregmap.gao-lab.org/) (Raza et al., 2022) was employed to procure GO information. Additionally, GPI modification motif (ω site for dissociation), the signal peptide, and hydrophobic plot were envisaged via the SignalP 4.1 Server (http://genome.cbs.dtu.dk/services/SignalP/), KYTE DOOLITTLE HYDROPATHY PLOT (http://gcat.davidson.edu/DGPB/kd/kyte-doolittle.htm), and the big-PI predictor (http://mendel.imp.ac.at/sat/gpi/gpi_server.html), correspondingly (Roudier et al., 2002; Niu et al., 2015).

### 2.8 RNA-seq datasets to explore the expression arrays of PtrCOBLs

Tissue preferential expression data of PtrCOBL genes in five different tissues (root, node, internode, mature expanding leaf, and young leaf) were adopted from PopGenIE (http://popgenie.org) and used to develop optical images (Sundell et al., 2015; Xu et al., 2017).

For further evaluation of the expression profiles of PtrCOBLs across abiotic stresses (cold, salt, drought, and heat), we selected previously reported transcriptome expression data of four stem samples, from PSDX (Poplar stem differentiating xylem) database (http://forestry.fafu.edu.cn/db/SDX) (Wang H. et al., 2021). For heat stress, plants were treated at 39°C for 7 days. For cold stress, plants were subjected to 4°C (night) and 12°C (day) for 7 days. For drought treatment, watering was withheld until the moisture of soil reached 0.1 m3/m3 and was maintained at the level of 0.06 – 0.1 m3/m3. For drought stress, plants were grown for 7 days after the initial leaf wilting point. For salinity stress, plants were treated with 100 mM sodium chloride solution for 7 days.

For expression analysis of PtrCOBLs in tension-wood formation, a publicly available transcriptome dataset was downloaded from NCBI (ID: GSE153793). To induce tension wood formation, 6-month old poplar plants were subjected to 90^0^ angle mechanical bending (vertical treatment), then tissues from stem differentiating xylem (SDX) were taken at 3 and 7 days post-treatment (Liu et al., 2021).

To investigate the PtrCOBL genes expression assay all through cambium growth and wood development (Sundell et al., 2017), transcriptome data with high spatial-resolution for the whole poplar plant was employed, including the cambium, secondary phloem, and xylem, available at ENA (ID: ERP016242) (http://www.ebi.ac.uk/ena/). Each replication tree has 25-28 samples collected from 15-mm-thick sections, underlying sections were taken across the cambial meristem and SCW developing xylem (http://aspwood.popgenie.org).

### 2.9 Plant materials, RNA extraction, and qRT-PCR analysis

Poplar (*Populus trichocarpa*) plants were endowed by the Zhejiang A & F University. Poplar seedlings (6 months old) were grown in the growth room at 16.0 h light, 20-25°C, and 70% air humidity. To examine expression patterns across different tissues, the plant samples were taken directly without treatment. The samples were snap-frozen in liquid nitrogen and kept at -80°C until additional analysis could be performed. In order to get consistent findings, all the experiments were conducted with three biological replicates, and three separate sets of samples were used in each experiment.

Plant total RNA was isolated out of roots, stems, and leaves using the RNAprep Pure Kit from TIANGEN Biotech (Beijing, China) following the given methodology. The PrimeScriptTM RT Reagent Kit (Takara Bio, Dalian, China) was used to isolate the first strand of cDNA after removal of genomic DNA. The qRT-PCR machine, ABI 7500 Real Time PCR System (Applied Biosystems, Foster City, CA, USA), was run using UltraSYBR Mixture (Low ROX) (CWBIO, Beijing, China). Each amplification reaction included 10.0 µL of 2x UltraSYBR Mixture (with ROX), 4.0 ng of cDNA template, and 0.2 µM of forward and reverse primers in a total volume of 20 µL. The qRT-PCR primers listed in (**Supplementary Table S1**) were all created using Primer Premier 5.0 (Premier Biosoft, Palo Alto, CA, USA) following these parameters for the reaction: 95°C for 2 min followed by 40 cycles of 95°C for 15 s and 60°C for 20 s. The 2^−ΔΔCT^ method was used to analyze the expression levels of PtrCOBL genes in different tissues. The relative expression variations of PtrCOBL genes were analyzed by using the PtrACTIN reference gene (**Supplementary Table S1**). The t-test was used to determine the statistically significant variations in the expression levels. Three separate experiments were conducted, and the results are shown as mean standard errors (p value: * p 0.05, ** p 0.01, *** p 0.001). Each sample was evaluated with three biological replicates.

## 3 Results

### 3.1 Identification of COBL genes and sequence analysis in poplar

In order to study the roles and physiogenomics of COBLs in poplar, an *in silico* study was carried out to identify the COBL-gene family members. The Basic Local Alignment Search Tool algorithms (BLASTp) and Hidden Markov Model (HMM) profile search were employed to retrieve the COBL members. Subsequently, these candidate COBL genes were further confirmed by SMART and NCBI-CDD tests, to confirm the genes possessing the (PF04833) domain. A total of 14 putative COBL genes were identified in the poplar genome v4.1 (*P. trichocarpa*) and were named as PtrCOBL1-PtrCOBL14 on the basis of their chromosomal locations. To clearly understand the characteristics of the COBL family in poplar, we analyzed the gene length, transcriptional sequence length, coding sequence (CDS) length, the position of the conserved domain, amino acid (AA) length, protein molecular weight (MW), grand average of hydropathicity (GRAVY), and isoelectric point (PI) of the proteins encoded by these genes (**Table 1**).

**Table 1:** Summary of COBL genes in Poplar.

Gene Name	Chr.	Strand	CDS (bp)	Protein Length	Mol. weight (KDa) Protein	pI	GRAVY	Cellular Localization	Potential ω-Site Position	CCVS Motif	Signal Peptide
PtrCOBL1	1	forward	1995	664	73.8821	8.72	-0.272	Extra- cellular	None	Yes	No
PtrCOBL2	4	forward	1362	453	50.74724	9.11	-0.145	Plasma Membrane	N(426)	Yes	Yes
PtrCOBL3	4	Reverse	1302	433	48.80238	8.92	-0.085	Plasma Membrane	S(411)	Yes	Yes
PtrCOBL4	4	forward	1335	444	49.66873	6.07	-0.113	Plasma Membrane	G(417)	Yes	Yes
PtrCOBL5	4	Reverse	1296	431	48.80097	8.22	-0.262	Lysosome	A(413)	Yes	Yes
PtrCOBL6	10	Reverse	1953	650	70.93764	5.24	-0.127	Extra- cellular	N(426)	Yes	Yes
PtrCOBL7	11	forward	1992	663	74.16447	8.87	-0.322	Extra- Cellular	S(634)	Yes	Yes
PtrCOBL8	12	Reverse	1317	438	48.73394	8.42	-0.049	Plasma Membrane	N(410)	Yes	No
PtrCOBL9	14	forward	1923	640	70.96131	5.28	-0.253	Plasma Membrane	S(618)	Yes	Yes
PtrCOBL10	15	forward	1374	457	50.98067	8.83	-0.107	Plasma Membrane	none	Yes	Yes
PtrCOBL11	15	Reverse	1299	432	48.40889	9	-0.185	Lysosome	N(408)	Yes	Yes
PtrCOBL12	15	Reverse	1320	439	49.43289	8.77	-0.117	Plasma Membrane	S(415)	Yes	Yes
PtrCOBL13	17	forward	1239	412	46.05201	9.49	-0.263	Extra- cellular	none	Yes	Yes
PtrCOBL14	17	forward	1245	414	46.14007	9.11	-0.206	Extra- cellular	none	Yes	Yes

Out of these 14 PtrCOBL genes, PtrCOBL13 was the shortest with 412 amino acids (AA) followed by PtrCOBL14 and PtrCOBL5 with 414 and 431 AA, respectively. PtrCOBL1 was the longest with 664 amino acids. The MW of PtrCOBL proteins was 22.200 to 68.678 kDa, the GRAVY of the proteins was – 46.052 (PtrCOBL13) to – 74.164 (PtrCOBL7), and PI was 5.24 (PtrCOBL6) to 9.49 (PtrCOBL13). Generally, identifying the molecular characteristics of genes is helpful in studying their specific biological roles.

**Figure 1:**
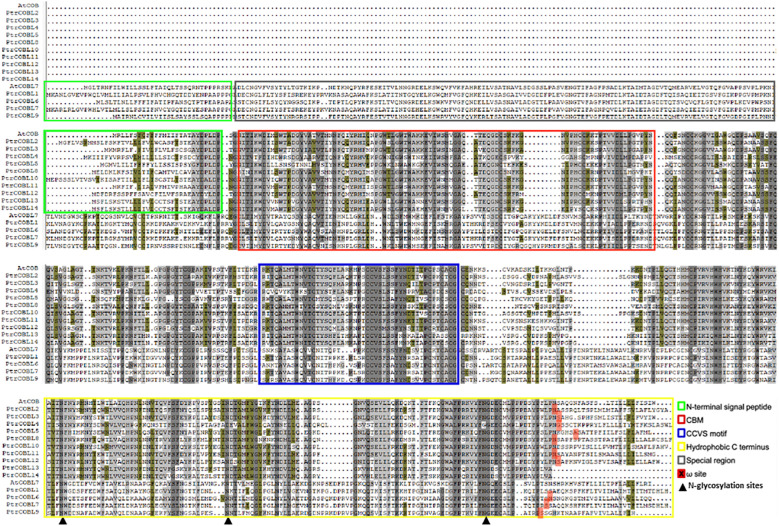
Multiple sequence alignment of COBL proteins. AtCOB-like group I proteins and AtCOBL7 group II proteins were aligned. The green boxes represent the N-terminal signal peptide, the red box denotes the cellulose binding motif, the blue box represents the CCVS-motif, the yellow box signifies the hydrophobic C-terminus, the gray box represents the special region found in group I, the solid red filled boxes epitomize ω sites (GPI attachment cleavage sites), and the triangle sign represents N-glycosylation sites in the sequence.

Based on the presence of the Pfam domain and similarity with query sequences, protein sequence alignment was carried out (**Figure 1**). Group I (similar to AtCOB) of *Populus* displayed the most conserved manner in their domains, while group II (similar to AtCOBL7) of *Populus* was the least conserved. Considering the number of *Populus* COBL genes in each group, group I was more conserved than group II. Poplar COBL genes overwhelmingly incorporated the CCV motif, N-terminal signal peptide, and ω site (GPI attachment cleavage site). PtrCOBL1 and PtrCOBL8 exceptionally showed the lack of an N-terminal signal peptide, while PtrCOBL1, PtrCOBL10, PtrCOBL13, and PtrCOBL14 were devoid of the ω site (**Figure 1**; **Table 1**).

### 3.2 Evolutionary and phylogenetic analyses of COBLs

**Figure 2:**
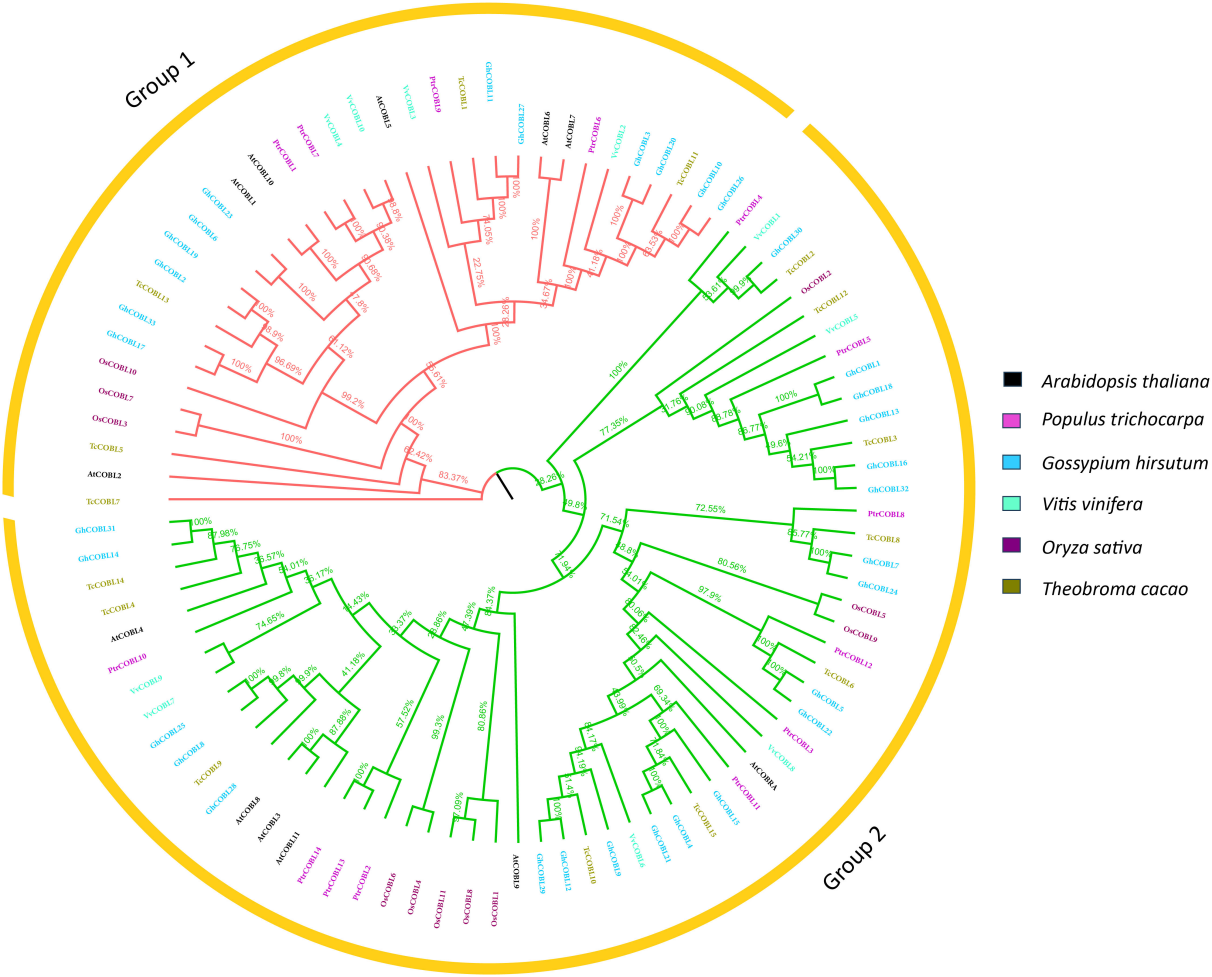
Phylogenetic analysis. The evolutionary relationship among six plant species, *Arabidopsis*, poplar, cotton, grape, rice, and cocoa. Muscle alignment was used to construct the unrooted, neighbor-joining tree.

To assess the evolutionary relationships among different plant species, a phylogenetic tree was constructed. For this purpose, full length protein sequences of *Arabidopsis*, poplar, rice, *Vitis vinifera*, *Theobroma cacao*, and cotton were retrieved. Using the multiple sequences alignment with the MEGA-X muscle program, a neighbor-joining tree was constructed (**Figure 2**). In this phylogenetic tree, protein sequences of 12 (*Arabidopsis thaliana*), 14 (*Oryza sativa*), 14 (*P. trichocarpa*), 15 (*Theobroma cacao*), 33 (*Gossypium hirsutum*), and 10 (*Vitis vinifera*) were used. Based on comparison with model plant *Arabidopsis* and analysis of COBL proteins’ diversity, these proteins were categorized into two subgroups, i.e., an AtCOB group and an AtCOBL7 group, designated as group 1 and group 2, respectively. Consistent with orthologs in *A. thaliana* and other species, the COBL family members were clustered into two subgroups GroupI and GroupII, phylogenetically related to AtCOBRA and AtCOBL7 in *A. thaliana*, respectively. Each subgroup had COBLs from six species, and a total of 6 genes from *Arabidopsis*, 4 genes from poplar, 12 genes from cotton, 3 genes from rice, 5 genes from *T. cacao*, and 4 genes from *V. vinifera* were found in group 1, whereas a total of 6 genes from *Arabidopsis*, 10 genes from poplar, 21 genes from cotton, 8 genes from rice, 11 genes from *T. cacao*, and 6 genes from *V. vinifera* were found in group 2. The phylogenetic analysis indicated that the COBLs were descendants of an ancient duplication that occurred even before the separation of monocots and dicots. By the high bootstrap values of the internal branches, it was possible to deduce that there are real homologs with likely similar functions.

### 3.3 Gene structure, conserved domain, and motifs of PtrCOBLs

**Figure 3:**
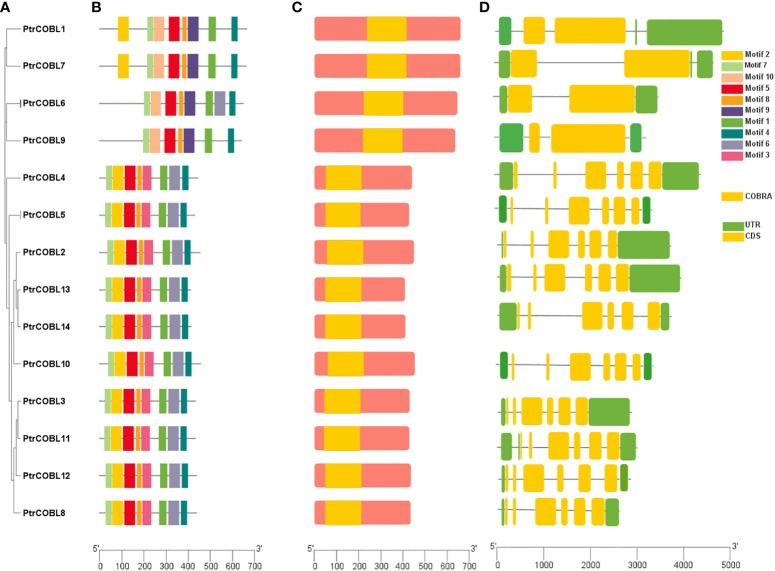
Motif and gene structure characterization. **(A)** Phylogenetic tree, **(B)** protein conserved motifs, **(C)** conserved domain analysis, with yellow indicating the *COBRA* domain, and **(D)** gene structure.

Gene structure and motif diversity is a mechanism that promotes the evolution of gene families. To structurally characterize PtrCOBLs, gene structure, conserved motif, and domain analyses were performed (**Figures 3A–D**). The total number of exons ranged from two to six in these genes, and this range varied between groups. As described earlier, COBL genes can be distinguished into two groups. The gene structure analysis showed that, despite being different gene lengths and exon positions, genes in the same group possess the same number of exons. This indicates the number of exons among the majority of PtrCOBL genes are highly conserved (**Figure 3D**). About 72% of genes occupying group II possess six exons, the largest number of exons, whereas PtrCOBL1, PtrCOBL6, PtrCOBL7, and PtrCOBL9 contain two exons, representing group I. This organization and structural conservation of genes supports the results of phylogenetic analysis (**Figure 3A**).

To identify motif composition and motif diversity among PtrCOBLs, the entire protein sequences of all the genes were submitted to the online MEME software. The average length of proteins appeared to be larger in group II in comparison with group I. Overall, 10 motifs were assigned and designated as motif 1-10 (**Figure 3B**). Interestingly, all the proteins contain the same number of eight motifs except PtrCOBL4. Motifs 9 and 10 are only found in PtrCOBL1, PtrCOBL2, PtrCOBL3, and PtrCOBL4, but motif 3 is missing in all these genes. Motif 6 is missing in PtrCOBL1, PtrCOBL2, and PtrCOBL4 but motifs 3 and 6 are only found in PtrCOBL 5 to 14. Notably, motifs 1, 4, 5, 7, and 8 were found in all the proteins, indicating these motifs are the essential components of PtrCOBL proteins. Domain verification tools indicated all the COBRA-*Like* genes of poplar contain the COBRA domain (**Figure 3C**).

### 3.4 Chromosomal localization

Through genome annotation, the COBL family genes were mapped to poplar chromosomes. Uneven distribution of genes on 8 chromosomes out of 19 chromosomes was observed in the COBL genes of poplar (**Supplementary Figure 1**). No gene was residing on the 2nd, 3rd, 5th, 6th, 7th, 8th, 9th, 13th, 16th, 18th, and 19th number chromosomes. All the 14 COBL genes were found asymmetrically distributed on just eight (1st, 4th, 10th, 11th, 12th, 14th, 15th, 17th) chromosomes. Only one gene was allocated to chromosomes 1, 10, 11, 12, and 14, while two genes were allocated to chromosome 2, followed by chromosomes 15 and 4 containing three and four genes, respectively. The PtrCOBL genes were sprinkled around different positions on the chromosomes: PtrCOBL1 was located from (44688589.44693374) on Chrom-1, PtrCOBL2 was located from (11002597.11006405) on chrom-4, PtrCOBL3 was located from (11006313.11008482) on chrom-4, PtrCOBL4 was located from (18547098.18551009) on chrom-4, PtrCOBL5 was located from (22495396.22499058) on chrom-4, PtrCOBL6 was located from (120146.123684) on chrom-10, and PtrCOBL7 was located from (16766946.16771575) on chrom-11. Similarly, PtrCOBL8 was located from (6499312.6501945) on chrom-12, PtrCOBL9 was located from (8418045.8421086) on chrom-14, PtrCOBL10 was located from (8383270.8386458) on chrom-15, PtrCOBL11 was located from (8386506.8389407) on chrom-15, PtrCOBL12 was located from (8390106.8393117) on chrom-15, PtrCOBL13 was located from (11038917.11042151) on chrom-17, and PtrCOBL14 was located from (11045798.11049523) on chrom-17.

The majority of PtrCOBL genes were located on chromosomes 4 and 15, which together harbor 50% of all the COBRA-*Like* genes from the *Populus trichocarpa* genome (four and three genes, respectively). Interestingly, we found that there were four PtrCOBL genes on chromosome 4 of the *Populus* linkage group (PtrCOBL2 to PtrCOBL5). In the same manner, three COBLs were on chromosome 15 (PtrCOBL10 to PtrCOBL12) of the linkage group, forming a relatively large tandem duplication region; it thus seemed that chromosomes 4 and 15 were a hot spot for studying the distribution of PtrCOBL genes. In addition, there were five fragment repeats located at the ends or near the ends of chromosomes 1, 10, 11, 12, and 14, respectively (**Supplementary Figure 1**).

### 3.5 Collinearity and synteny analysis among plant species

**Figure 4:**
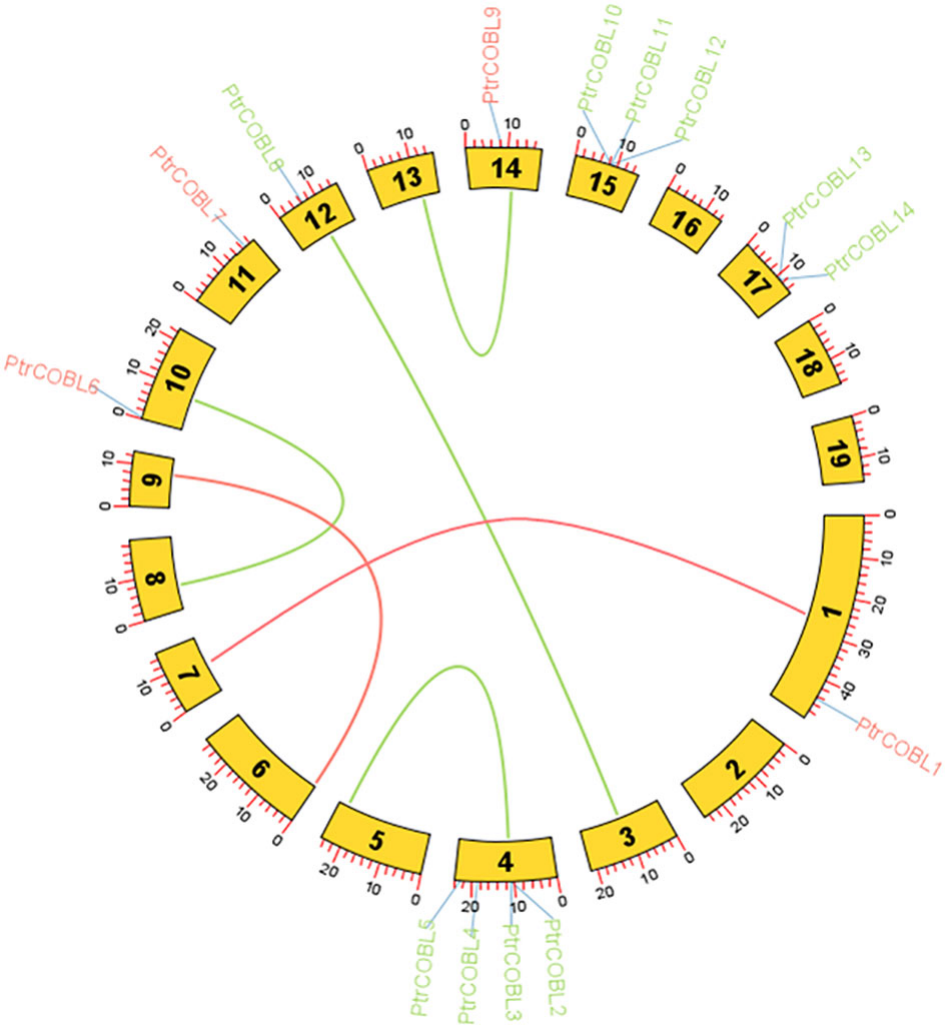
Fragment duplication of the poplar COBL genes. The outer circle blocks represent the chromosome numbers and gene density on the chromosome; the red and green lines represent the fragment duplicate gene pair. The length of each arc corresponds to the length of the chromosome (Mb).

Circos analysis was employed to identify duplication events within the poplar genome. Three sorts of events were observed in plant species gene duplication, including tandem (a duplicated chromosomal region within 200 kb containing two or more genes and occurring adjacent to the original), segmental (genes located on duplicated chromosomal blocks 1 kb to 400 kb that occur at multiple locations throughout the genome), and whole genome duplication (the process which generates additional copies of the genome). The gene duplication events may influence the gene function diversity among plant species. In total, six paralog pairs were observed, where PtrCOBL1 was a paralog pair of PtrCOBL7 and PtrCOBL12 was a paralog pair of PtrCOBL3. A third paralog pair comprised PtrCOBL4 and PtrCOBL5. Likewise, PtrCOBL6 and PtrCOBL9 formed a fourth paralog pair, and the fifth represented PtrCOBL8 and PtrCOBL10. The sixth paralog pair was PtrCOBL13 and PtrCOBL14 (**Figure 4**; **Supplementary Figure 1**).

**Figure 5:**
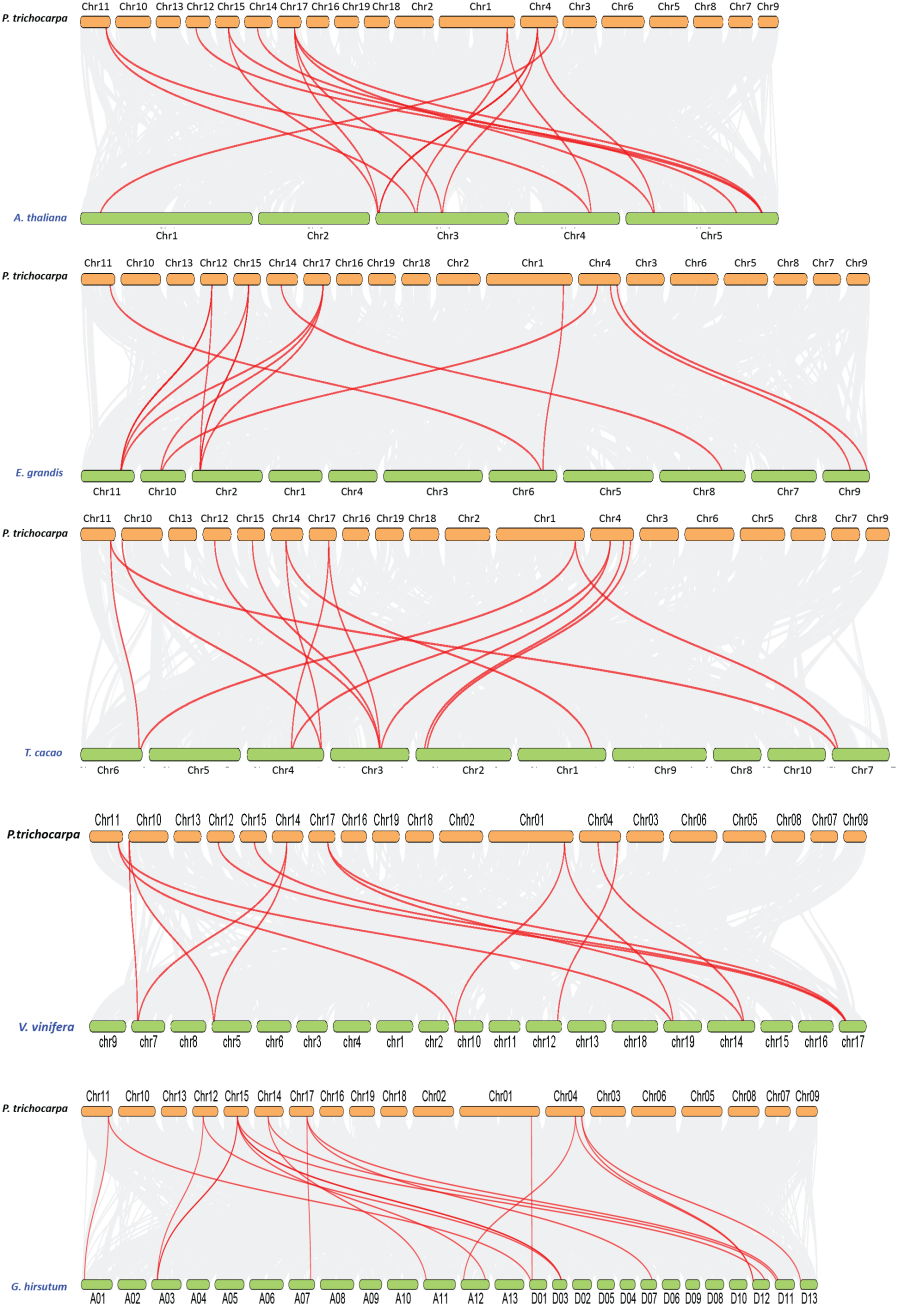
Collinearity of the COBL genes from poplar and other five plant species. Gray lines in the background indicate the collinear blocks within poplar and other plant genomes, while the orthologs between two species are connected with red lines.

Tandem duplication events were observed in two paralogous gene pairs, i.e., PtrCOBL4/PtrCOBL5 and PtrCOBL13/PtrCOBL14, whereas a series of segmental duplication events were observed in this study that likely increased the number of COBL genes in poplar as compared to *Arabidopsis*. PtrCOBL1 and PtrCOBL7 experienced segmental duplication. Similarly, PtrCOBL6 and PtrCOBL9 were also tandemly duplicated, as were PtrCOBL8 and PtrCOBL10. Furthermore, PtrCOBL3/PtrCOBL12 also indicated segmental duplication event occurrence (**Figure 4**). In order to further study gene duplication, we constructed five PtrCOBL comparative synteny maps (**Figure 5**). Among these maps, the strength of correlation with PtrCOBL genes in ascending order was given as: *Arabidopsis* (17), T. *cacao* (15), grape (14), *Eucalyptus* (15), and cotton (18). Comparative collinear mapping between monocotyledons and dicotyledons depicted that the tandem duplication events and the segmental duplication events may not only be the primary mechanism of gene family expansion in plant evolution but may also make a significant contribution to the diversity of gene families.

### 3.6 Cis-acting elements of promotor and GO annotation

**Figure 6:**
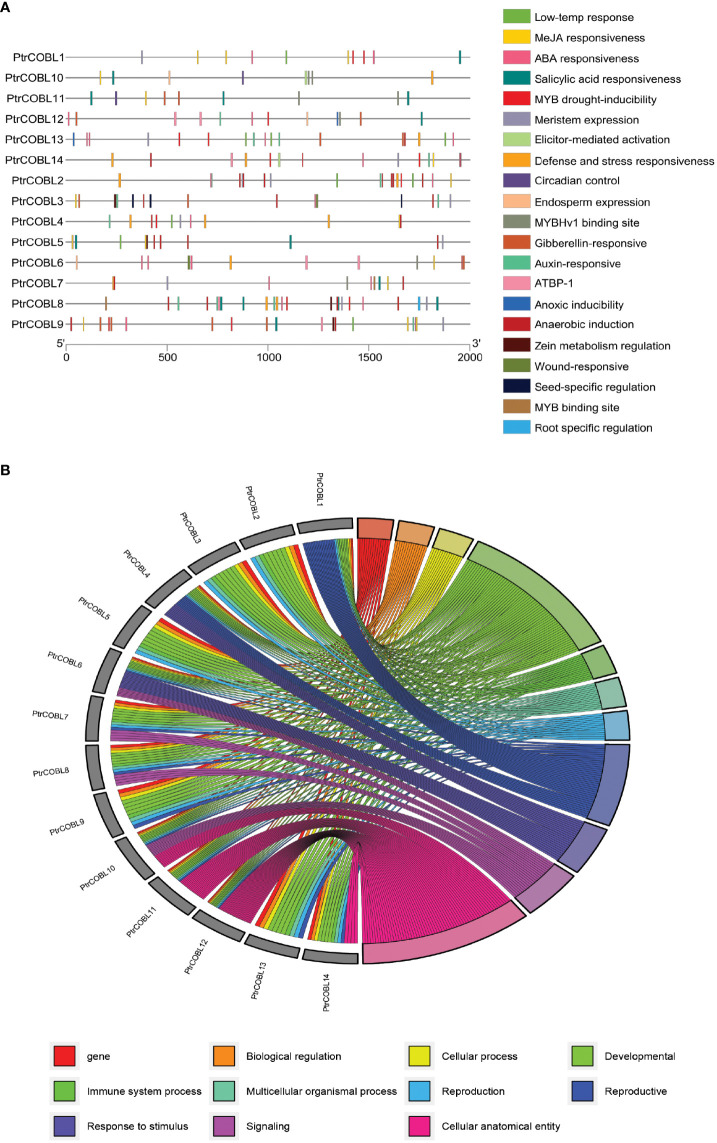
Upstream cis-regulator elements and gene ontology analysis: **(A)** Predicted cis-elements in COBLs promoters. Diverse colors were used for representing different cis-elements, as given on the right side. **(B)** Gene ontology annotation of COBLs in poplar; different colors represent different biological, molecular, and cellular processes.

The studies on the non-coding regions of genes are becoming of prime importance. Cis-acting regulatory elements (CRE) in the promoter region of the genes influence transcription and thus regulate the functions of the genes (**Figure 6A**). The upstream region (of 2000 bp) from the site of initiation (ATG) was uploaded to the PlantCare database. *In silico* promotor analysis resulted in the identification of 21 CRE sites in the COBL genes; along with common CREs there were plenty of cis-elements identified related to low temperature response (LTR), MeJA responsiveness, ABA responsiveness, Salicylic acid responsiveness, Gibberellin responsive, Auxin responsive, MYB binding site, MYB drought inducibility, MYBHv1 binding site, AT rich DNA binding protein (ATBP-1), anoxic inducibility, anaerobic induction, stress responsive, and elicitor-mediated activation and defense. Importantly, regulatory motifs for meristem-expression, wound responsiveness, seed specific regulation, root specific regulation, endosperm expression, circadian control, and zein metabolism regulation were also identified (**Supplementary Table S2**). Out of all these CREs, ABA and auxin-related regulatory motifs were abundantly found in the COBL genes of poplar. Conjointly, the results indicated that the functional expression of PtrCOBL genes is controlled by cis-regulatory elements linked to plant growth and development, plant stress response, and hormonal response.

To brainstorm functions of COBL family genes, gene ontology (GO) annotation and enrichment analysis was enacted on the basis of their biological processes (BP), molecular functions (MF), and cellular component (CC) sets. The results revealed that several GO terms were enriched in the BP, MF, and CC (**Figure 6B**). The 14 COBL proteins were assigned with 48 GO terms, where BP terms were extensively enriched followed by MF and CC. Among BP enrichment, analysis suggested that 25 terms were highly enriched: cellulose microfibril organization (GO:0010215), cell growth (GO:0016049), plant-type cell wall organization or biogenesis (GO:0071669), cellular component organization or biogenesis (GO:0071840), extracellular matrix organization (GO:0030198), extracellular structure organization (GO:0043062), cellular component biogenesis (GO:0044085), cellular component assembly (GO:0022607), external encapsulating structure organization (GO:0045229), and single-organism cellular process (GO:0044763) were abundant. Moreover, vegetative to reproductive phase transition of meristem (GO:0010228), response to temperature stimulus (GO:0009266), response to osmotic stress (GO:0006970), and response to abiotic stimulus (GO:0009628) were also among the annotated terms. In the MP enrichment, polysaccharide binding (GO:0030247), chaperone binding (GO:0051087), carbohydrate binding (GO:0030246), and hydrolase activity, hydrolyzing O-glycosyl compounds (GO:0004553) were enriched. In the CC set, anchored component of membrane (GO:0031225), intrinsic component of membrane (GO:0031224), membrane (GO:0016020), membrane part (GO:0044425), and cell junction (GO:0030054) were enriched. This GO analysis indicated that COBL genes are extensively involved in plant growth and development related functions at BP, MF, and cellular levels.

### 3.7 Tissue-specific and qRT-PCR expression profiles of COBL genes

**Figure 7:**
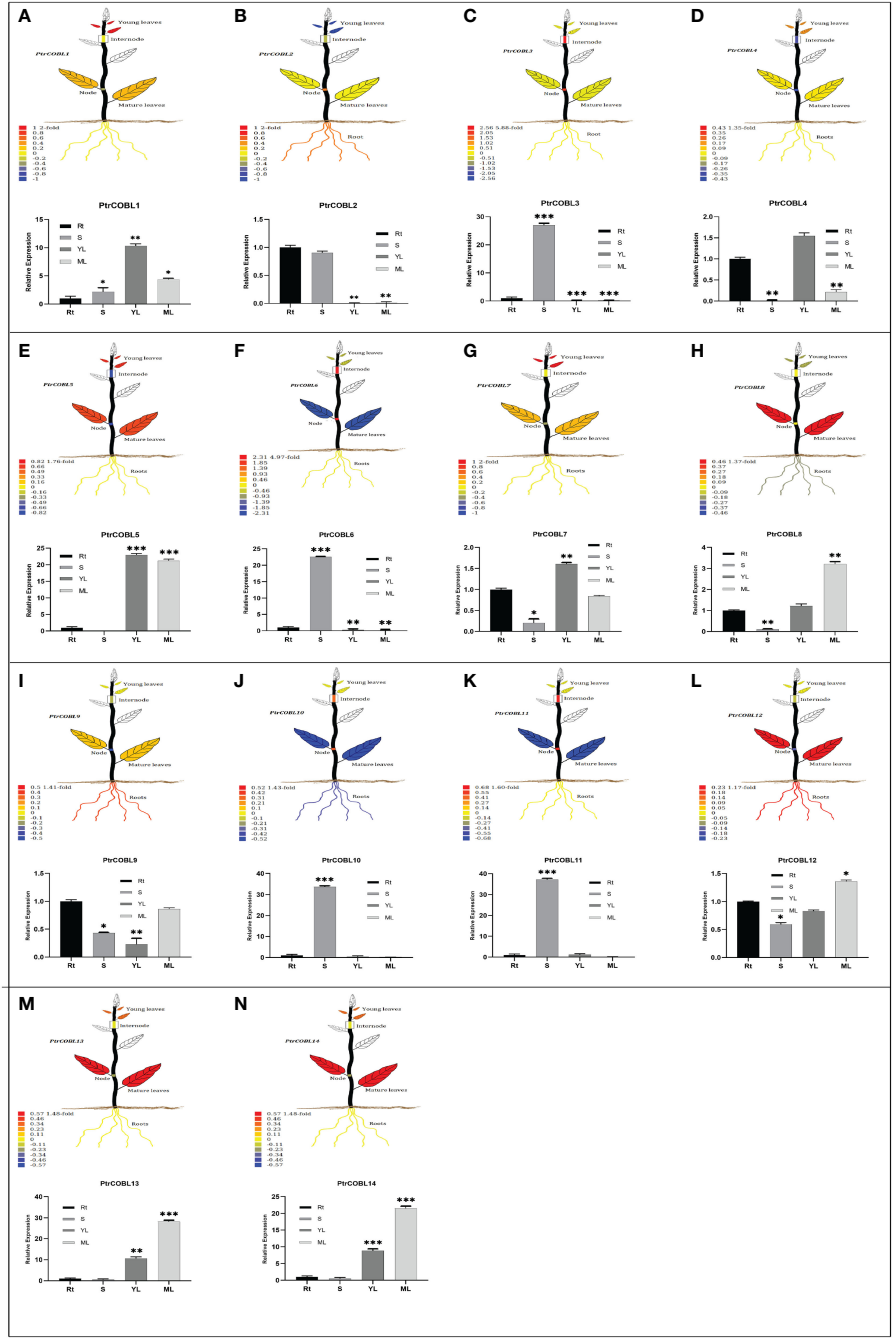
Tissue-specific expression profiles of PtrCOBLs. The PopGenIE v3 database (accession number: GSE6422) was utilized to generate visual plant images. Different colors of the plant organs indicate the expression abundance of the respective genes **(A-N)**. The bar graphs were generated employing RT-qPCR to verify relative expressions. The data represent the means ± SE from three independent experiments, * p < 0.05, ** p < 0.01, *** p < 0.001.

To investigate the potential functions of the PtrCOBL genes in the developmental processes of *P. trichocarpa*, the tissue-specific expression pattern of PtrCOBLs was analyzed (**Figures 7A–N**). The visual representations of plant tissues were created using the Plant Genome Integrative Explorer. Herein, PtrCOBL1, 4, 5, and 7 were highly expressed in young leaves (YL) followed by PtrCOBL13 and 14, where gene expression was abundant in YL. PtrCOBL3, 6, 10, and 11 were found highly expressed in stems (S) followed by PtrCOBL2, where gene expression was abundant in stem (node and internode) zones. Similarly, PtrCOBL5, 8, 9, 12, 13, and 14 followed by PtrCOBL7 were found to be highly expressed in mature leaves (ML). PtrCOBL2, 9, and 12 were discovered to be predominantly active in the root area.

Then, RT-qPCR was used to validate the accuracy of microarray data results. Overall, microarray data results were corroborated by RT-qPCR, but there were some inconsistent expression patterns found in the roots, stems, and leaves. It is noteworthy that PtrCOBL1, 7, 9, and 12 in stem tissues and PtrCOBL8 in YL showed slightly different expression as compared to RT-qPCR results. PtrCOBL1, 7, 9, and 12 demonstrated more expression in stem area, whereas PtrCOBL8 expressed more in YL as compared to the microarray data, which may be due to variations in their testing materials and conditions (e.g., environmental factors and sample gathering intervals).

### 3.8 Whole-genome mining of miRNAs directing COBL genes

**Figure 8:**
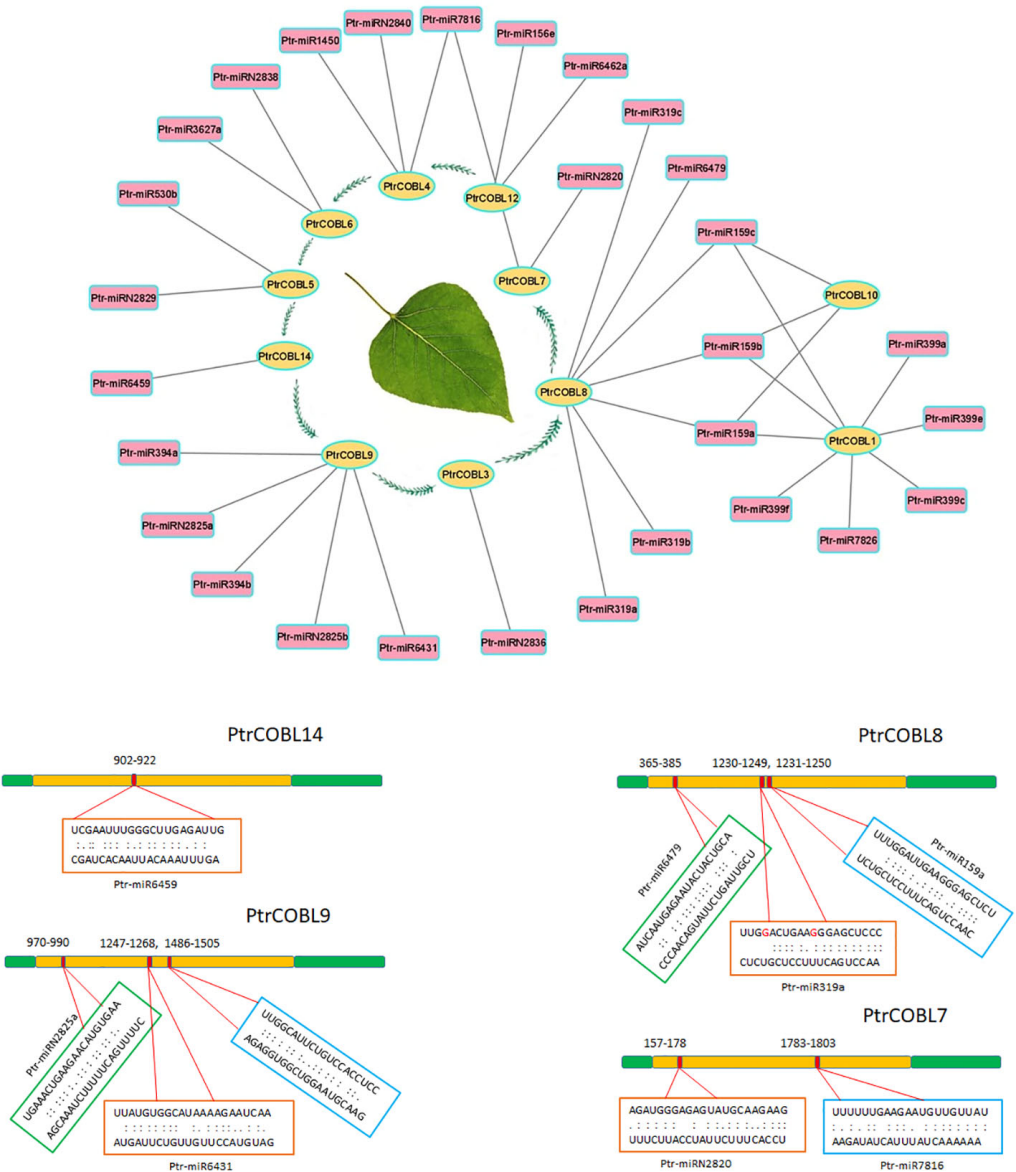
mi-RNA targeting network and their target sites in poplar COBLs. Genes are located in the oval shapes, while targeting miRNAs are in the rectangular shapes. Lines connect different genes to their putative miRNAs. Multiple miRNAs sharing the same target site are represented once.

Numerous studies have reported a significant impact of micro-RNAs on fine-tuning plant growth and development. To explore the miRNA-targeted post-transcriptional modification of COBL genes, 29 putative miRNAs (belonging to 20 different families) were identified regulating 11 genes (**Figure 8**; **Supplementary Figure 2**). To provide insight into miRNA regulation, (**Figure 8**) depicts the target sites on PtrCOBL7, PtrCOBL8, PtrCOBL9, and PtrCOBL14. Detailed report of all the miRNA associated genes and their targeting sites are presented in **Supplementary Figure 2**. Global analysis showed that PtrCOBL8 was targeted by seven miRNAs (Ptr-miR159a, b, c; Ptr-miR319a, b, c; Ptr-miR6479). In the same manner, PtrCOBL9 was targeted by Ptr-miR6431, Ptr-miR2825, and Ptr-miR394. PtrCOBL4 was targeted by Ptr-miR7816, Ptr-miR2840, and Ptr-miR1450. PtrCOBL1 was found to be directed by Ptr-miR159, Ptr-miR399, and Ptr-miR7826. PtrCOBL12 was targeted by Ptr-miR156 and Ptr-miR6462; PtrCOBL7 was targeted by Ptr-miR2820 and Ptr-miR7816. Interestingly, Ptr-miR159 targeted the most, nine sites of three different genes, i.e., PtrCOBL1, 8, and 10, whereas PtrCOBL5 was targeted by Ptr-miR530 and Ptr-miR2829, while PtrCOBL6 was targeted by Ptr-miR2838 and Ptr-miR3627. Ptr-miR7816 targeted two different genes, PtrCOBL4 and PtrCOBL7. Furthermore, PtrCOBL3 and PtrCOBL14 were found to be targeted by only one miRNA. Therefore, validation of the expression profiling of these predicted miRNAs and their target genes is required to evaluate their biological functions in the poplar genome.

### 3.9 Expression profiling and co-expression network analysis for abiotic stress

**Figure 9:**
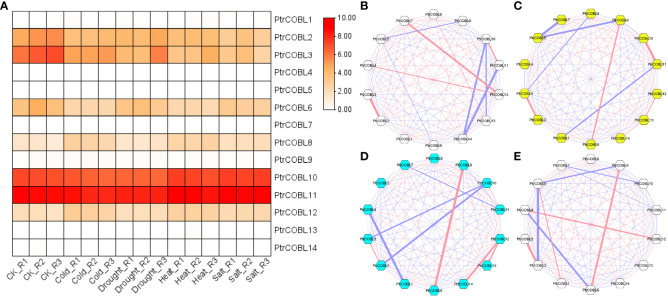
Expression profiling and co-expression network analysis for abiotic stress. Heat map of expression patterns. **(A)** The co-relation networks of 14 COBL genes in **(B)** cold, **(C)** heat, **(D)** salt, and **(E)** drought were analyzed based on Pearson correlation of the selected COBL genes obtained from transcriptomic data. The red and blue edges show positive and negative correlations, respectively. However, the thickness of each edge shows the strength of the correlation for each pair.

The PtrCOBL genes were surveyed against multiple abiotic stimuli to investigate their ability to withstand stress. Employing FPKM values (publicly available RNAseq data), expression patterns of COBL genes toward the endurance of abiotic stresses in poplar was observed. Almost half of the genes were found to be expressed in all abiotic stresses, i.e., drought, heat, cold, and salt stress (**Figure 9A**). PtrCOBL10 along with PtrCOBL11 showed the highest expression among all the genes, consistent with all the stresses. Notably, PtrCOBL10 demonstrated higher expression in heat and salt stress compared with cold and drought stress. Conversely, PtrCOBL11 showed perpetually higher expression among all the abiotic stresses. The same went for PtrCOBL2 and PtrCOBL3, where PtrCOBL3 showed high expression in cold and drought stress in comparison to heat and salt stress. PtrCOBL6 demonstrated constant expression except in the drought stress, where higher expression was observed, suggesting PtrCOBL6 expression was related to drought response. PtrCOBL12 also showed an unswerving expression pattern. Contrastingly, PtrCOBL8 exhibited the least expression in abiotic stresses among all the genes. These results suggested that COBL genes take part in abiotic stress response mechanisms.

Having said that, to identify hub genes, the co-expression network analyses were performed based on the correlation coefficient of their respective expression data. Co-expression network analysis showed positive as well as negative correlations among all the abiotic stresses. In the case of cold stress, 46 gene pairs were positively correlated while 45 gene pairs were negatively correlated (**Figure 9B**). In regard to drought stress, 51 gene pairs were found to be positively correlated while 40 gene pairs were found to be negatively correlated (**Figure 9E**). As far as heat stress was concerned, there were 44 positively correlated gene pairs and 47 negatively correlated gene pairs. (**Figure 9C**). Moreover, in salt stress, 53 gene pairs were found to be negatively correlated while 38 gene pairs were found to be positively correlated (**Figure 9D**). Considering the high expression and large number of correlation links, hub genes were decided. Interestingly, PtrCOBL2 and PtrCOBL3 turned out to be hub genes in each stress group.

The cold stress network showed that PtrCOBL2 and PtrCOBL3 shared a strong positive correlation. Likewise, PtrCOBL10 and PtrCOBL11 were linked with a great positive correlation. In contrast, PtrCOBL10 had a strong but negative correlation with PtrCOBL13 and PtrCOBL14, and similarly PtrCOBL11 also exhibited a strong but negative correlation with PtrCOBL14. All these correlation links were consistent with expression patterns shown in the heatmap. In the case of heat stress, PtrCOBL2 and PtrCOBL3 shared a strong and positive correlation and, similarly, PtrCOBL10 and PtrCOBL11 were also found to share strong and positive correlation. On the flip side, some interactions were not found to be in cooperation in the expression data, e.g., PtrCOBL3 had a negative correlation with PtrCOBL8 while in the salt stress network, PtrCOBL12 had a positive correlation with PtrCOBL13 and PtrCOBL14. In the same line, PtrCOBL10 had a negative correlation with PtrCOBL2 and PtrCOBL3. This may serve as a foundational guide for further research into how the PtrCOBL genes interact.

### 3.10 Expression profiling and co-expression network analysis for wood formation

**Figure 10:**
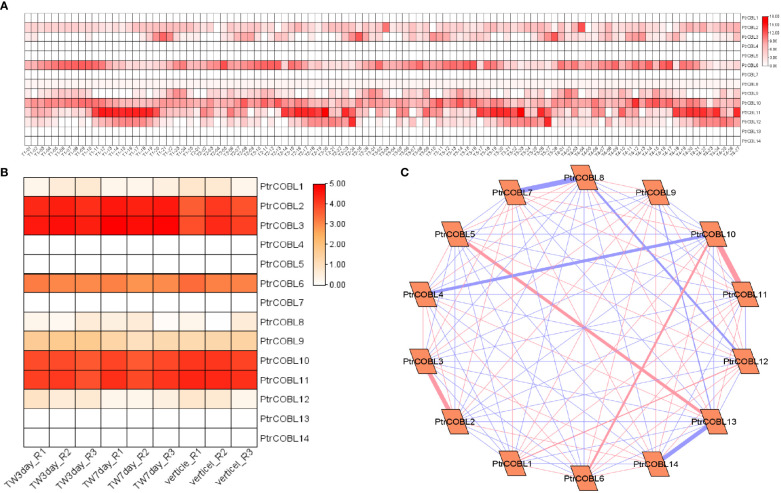
Expression profiling and co-expression network analysis for wood formation. **(A)** Heat map of the expression patterns of COBLs in all the wood forming tissues. **(B)** Heatmap of the expression patterns of COBLs in tension-wood forming tissues. **(C)** The co-relation networks of 14 COBL genes in tension-wood formation were analyzed based on Pearson correlation of the selected COBL genes obtained from transcriptomic data. The red and blue edges show the positive and negative correlations, respectively. However, the thickness of each edge shows the strength of the correlation for each pair.

Transcriptome data (publicly available) were used to investigate the putative functions of PtrCOBL genes in wood formation, where the expression of PtrCOBL genes was as follows: PtrCOBL1 along with PtrCOBL12 displayed moderate expression. On the flip side, PtrCOBL1, 4, 5, 7, 13, and 14 presented minute levels of expression in early xylem, mature xylem, phloem, pith, and other wood fiber tissues (**Figure 10A**). COBL 2, 3, 6, 9, 10, 11, and 12 showed a good level of expression in all the tissue sections. Overall, the entire list of COBL genes showed expression in xylem, cambium, and phloem development tissues. This analysis gave an approximate measure of the xylem area containing living vessels, fibers, and other tissues taking part in stem growth and plant girth (**Figure 10A**). To further explore the role of PtrCOBLs in developing tissues, expression profiles were investigated under tension-wood formation. Interestingly, the majority of COBLs presented good expressions; COBL 8 and 12 showed a minute level of expression whereas COBL 2, 3, 6, 9, and 10 and PtrCOBL11 divulged higher levels of expression. This expression indicated a pivotal role of COBLs in tension wood developing tissue (**Figure 10B**).

Moreover, co-expression network analysis was done to investigate the interactions among all COBL genes during tension wood development (**Figure 10C**). PtrCOBL5 and PtrCOBL13 showed strong positive interaction; likewise PtrCOBL6 and PtrCOBL10 showed strong and positive interaction. PtrCOBL4 and 10 showed strong but negative interaction, as PtrCOBL7 and 8 showed negative interaction, whereas PtrCOBL 8 with 12 and COBL5 with 13 also showed negative interaction. Similarly, PtrCOBL9 with 13 showed negative interaction. Interestingly, PtrCOBL13 and 14 were found to be negatively interacted. PtrCOBL12 and 13 showed a total of 100 interactions with the majority being positive ones. We identified hub genes based on the large number of correlation hits and high expression. Intriguingly, two genes (PtrCOBL2 and PtrCOBL3) stood out as hub genes, suggesting that these two genes play an important role in the development of wood in the poplar plant.

## 4 Discussion

**Figure 11:**
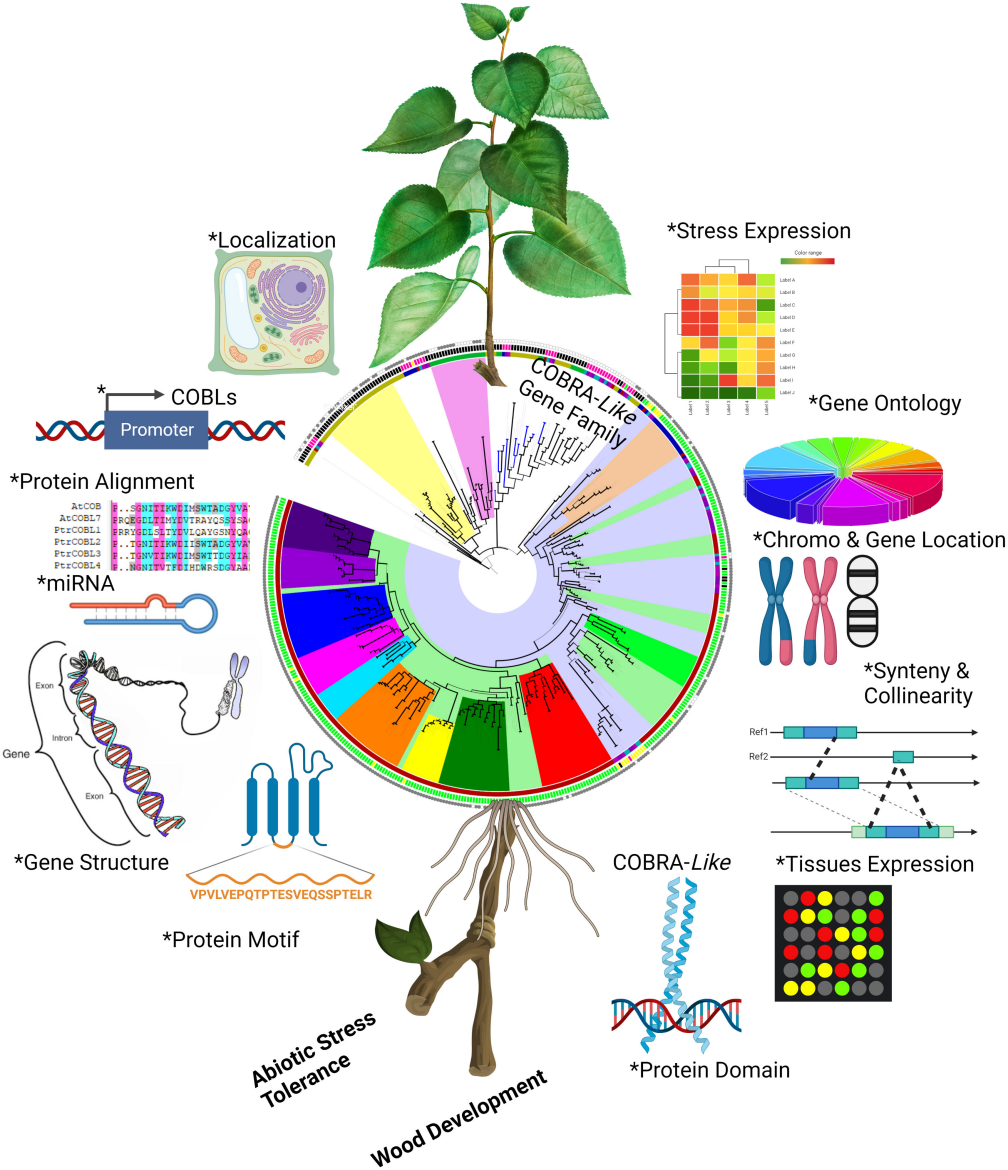
Summary of the comprehensive study of COBL genes in poplar. Asterisk symbol indicate different analyses were performed to get insight into the characterization of PtrCOBL genes. The figure was constructed using the BioRender tool.

The world’s population is expected to reach roughly 9.2 billion by 2050, and the current rate of increase in plant yield and biomass is not enough to fulfill their demands (Zaheer et al., 2021). It is generally known that abiotic stress hinders plant growth, development, and production, and climate change is projected to worsen severe weather events, making the situation even more precarious (Liu et al., 2015). In this scenario, the importance of forest trees has already increased: tree plants are simply a lifeline for earth. To develop more rigorous and resilient plants we need to improve our comprehension about the rudimentary processes, functions, and evolutionary mechanisms in plants (Leng et al., 2021). The advent of next-generation sequencing techniques has revolutionized the plant genomic landscape by making data from an endless variety of dimensions available. There is now the possibility of doing more accurate genome-wide analysis because to the availability of recently re-sequenced high-quality poplar genome assemblies (Liu et al., 2015; Sangi et al., 2021) (**Figure 11**).

### 4.1 Identification and evolution

The COBRA gene family has been reported in many other plant species. Even the moss *Physcomitrella patens* has been shown to contain members of the COBRA family. The ancestor of *Arabidopsis* already had members of this family (Roudier et al., 2002). There are 14 members in group I of the COBL genes in poplar, and they are most closely related to the COB genes in the *Arabidopsis* genome. Whilst group II proteins, which consist of four members, are alike to the AtCOBL7 subfamily, they are longer in size than subfamily I proteins. When comparing groups I and II, the main difference is the 170 more N-terminal amino acids found in group I (Brady et al., 2007). The differentiation between the two clades is supported by the differences in gene structure and the number of exons and introns. After the break between eurosid I (*Populus*) and eurosid II (*Arabidopsis*), there may have been divergence that led to this (Roudier et al., 2002). The COBL genes encode for proteins that are anticipated to have a cellulose binding site, a Cys-Cys-Val-Ser motif (CCVS), a possible N-glycosylation site, and an N-terminal peptide signal. Cleavage of the proteins at the GPI anchor modification sites (ω sites) and subsequent addition of the GPI anchor structure through amide bonding occurs at the c-terminus (Roudier et al., 2002; Niu et al., 2015) (**Figure 1**; **Table 1**). Furthermore, phylogenetic research revealed that both monocot and dicot COBLs are present within the two groups (**Figure 2**). This study provides more evidence that members of the COBL family are descended from a duplication that happened prior to the split between monocots and dicots (Tuskan et al., 2006; Ye et al., 2009). The number of COBL genes is larger in dicots because of repeated gene duplications. The very similar gene structures across PtrCOBL genes provide strong evidence for the extensive conservation of this gene family (**Figure 3**). Weak selection pressure and a higher intron abundance were also thought to have allowed introns to evolve into unique functional roles (Zaheer et al., 2021).

### 4.2 Gene structure and intron exon genome expansion

COBL genes play a vital role in cell growth, wood fiber, and cellulose synthesis. The function and biological significance of the COBL proteins in a wide variety of plants, *Arabidopsis*, brassica, soyabean, rice, cotton, wheat, *L. Chinense*, and *Populus*, have been the subject of study (Dai et al., 2009; Ye et al., 2009; Niu et al., 2015; Sangi et al., 2021; Yang et al., 2021; Zaheer et al., 2021; Qiu et al., 2023). There has not been a methodical and comprehensive study of COBL genes in poplar. Studies on eukaryotic diversification have shown extensive intron gain or loss. An identical exon–intron configuration was found while analyzing duplicated genes (**Figure 3**). We discovered that all COBL genes have the same number of introns except COBL 1, 7, 6, and 9 (paralog pairs; 1-7, 6-9), which have only one intron, indicating that COBL protein in poplar was recently evolved with fewer introns, corroborating that the number of introns has been decreasing with time, meaning that more recently developed species have fewer introns than more ancient ones (Zaheer et al., 2021). In the same manner, all the COBL have same number of exons (CDS), except COBL 1, 7, 6, and 9. Notably the COBL1, 7, 6, and 9 group is alike and the COB group is alike (**Figure 3A**). Significant insertion/deletion events may affect the exon–intron structure. In plant evolution, introns have a major impact on the development of new species (Roudier et al., 2002; Brady et al., 2007).

### 4.3 WGD collaborated to COBL gene family expansion

Evidence suggests that, compared to monocots, the number of family members in eudicots has expanded. Several potential explanations have been proposed for this expansion, including whole-genome duplication and segmental duplication (Tang et al., 2008). Whole-genome duplication (WGD) occurred twice in the poplar genome, first at 8-13 Mya and again around 60-65 Mya (Tuskan et al., 2006; Sundell et al., 2015). Only two paralogous gene pairs (PtrCOBL4/PtrCOBL5 and PtrCOBL13/PtrCOBL14) were found tandemly arranged, while all the other paralogous genes pairs were scattered on the different chromosomes (**Figure 4**; **Supplementary Figure 1**), revealing the expansion of the COBL gene family in poplar, primarily due to segmental duplication rather than singletons or tandem duplication (Bano et al., 2021; Yang et al., 2021). This confirms prior findings that segmental duplication has been predominated in the poplar genome. Based on our data, we were able to determine that only two paralog pairs originated from the first WGD and four paralog pairs originated from the second WGD (Cheng et al., 2021; He et al., 2021). We hypothesize that the recent WGD duplication may be the primary mechanism for the growth and functional diversity of COBL genes in poplar (**Figures 4**, **5**). To analyze the non-synonymous/synonymous mutation ratio of PtrCOBL genes, the Ka/Ks ratios were determined. Pseudogenes emerge from neutral selection when the Ka/Ks ratio is 1, purifying selection favors duplicated genes with a Ka/Ks ratio below 1, and positive selection of rapid evolution is shown with a Ka/Ks ratio above 1. All the COBLs have Ka/Ks ratios below 1, suggesting that the COBL gene family was subject to purifying selection over the course of evolution (Niu et al., 2015; Bano et al., 2021; Kabir et al., 2022). The Ka/Ks ratios for PtrCOBL12-PtrCOBL3 and PtrCOBL8-PtrCOBL10 were the lowest among the paralogous genes, indicating these genes were able to keep their original roles despite their shorter divergence dates. The highest Ka/Ks value and very divergent expression patterns were found for the paralog pair PtrCOBL13-PtrCOBL14, providing strong evidence for their functional divergence after duplication (Sangi et al., 2021; Yang et al., 2021).

### 4.4 Cellular localization and promotor analysis of PtrCOBLs

The subcellular location of genes play a significant role in determining their function (Wang et al., 2022). The COBRA-*Like* proteins have highly conserved structures. Evidence indicates that, similar to COBL proteins, cellulose synthase complexes (CSCs) are assembled in the endoplasmic reticulum (ER) or the Golgi apparatus. The N-terminal signal peptide and C-terminal GPI anchoring motif of COBRA-*Like* proteins are hypothesized to direct the protein to the outer leaflet of the plasma membrane (McFarlane et al., 2014; Sangi et al., 2021). It is unclear whether proteins encoded by COBRA genes that lack any of these patterns are functional. After a protein has been localized to the outer leaflet of the plasma membrane, the GPI anchor motif is usually disrupted (Zaheer et al., 2021). It is thus likely that COBRA’s ability to bind cellulose is crucial for its proper localization at the plasma membrane-cum-cell wall interface (Roudier et al., 2002; Zaheer et al., 2021). The majority of genes showed a strong localization signal in Golgi apparatus coupled with plasma membrane and/or vacuole, in agreement with the results (Qiu et al., 2023). Particularly PtrCOBL2 is dual-targeted to plasma membrane and/or vacuoles whereas PtrCOBL4 and PtrCOBL13 are extracellular- and vacuole-specific (**Table 1**).

Gene expression control occurs primarily via transcriptional regulation, which requires interaction between transcription factors and promoter binding sites (Wang et al., 2022). Specific and consistent cis-acting elements in promoter regions are required for different external signals to activate inducible promoters; for example, auxin-induced promoters typically contain cis-acting elements of the AuxRE, and light-induced promoters typically contain cis-acting elements rich in the AT-rich motif, the I-box, the G-box, and the GT1-motif (Kabir et al., 2022; Wang et al., 2022). Similarly, drought-induced promoters included the cis-acting elements CACG and CATGTG. We isolated sections of potential genes’ upstream promoters for this analysis (**Figure 6A**). Rigorous appearance of MeJA, Auxin, SA, and ABA response elements in COBL promotor regions implied their involvement in stress and plant development (Yu et al., 2021; Qiu et al., 2023). Phytohormones have been proven in previous research to ensure that plant cell walls remain unaltered. MeJA has a mediatory role in the regulation of genes in response to plant damage. Auxin controls cell wall thickness by stimulating membrane permeability (Ye et al., 2009). Strawberry fruit softens and ripens with the help of ethylene, which controls the production of pectin (Iqbal et al., 2022; Kabir et al., 2022). Interestingly, meristem and wound response related cis-elements prove COBLs’ direct role in wood development and tension wood forming tissue in poplar (**Supplementary Table S2**). In plants, the TATA-box has been shown to have a role in the regulation of transcription via the mediation of miRNA (Kabir et al., 2022).

### 4.5 Global analysis of micro-RNAs in poplar

MicroRNAs (miRNAs) are a class of single-stranded, non-coding RNAs that play a role in post-transcriptional gene regulation (Wang et al., 2022). Genome-wide investigation has espied 29 miRNAs of 20 families (**Figure 8**; **Supplementary Figure 2**) belonging to 108 total miRNA families currently reported in *P. Trichocarpa* (Guo et al., 2022). Using our methodology, miRNA specific target sites were determined in the genes of PtrCOBL family (**Figure 8**; **Supplementary Figure 2**). Most of these miRNAs have been reported with high expression in xylem and leaf area, indicating their key role in plant growth and development and stress response. For instance, many plant families, including angiosperms, mosses, and lycopods, share a highly preserved version of MIR159 because of its essential role in plant growth, with high expression in xylem tissues and leaf (Guo et al., 2022; Kabir et al., 2022). The miR159 targets the MYB and NAC genes to fine-tune the juvenile development. miR159 interacts with ABI5 shape the vegetative development (Guo et al., 2021). Ptr-mRNA319, Ptr-miR1450, and Ptr-miR6462 are involved in the regulation of secondary growth in plants, with high expression in xylem (Wang R. et al., 2021). Ptr-miR6479 is involved in oxidation reduction and cell development. Ptr-miR6431 is reported to be ABA responsive and involved in stress and growth-related functions. Ptr-miR394, Ptr-miR156, Ptr-miR530, Ptr-miR6459, and Ptr-miR3627 have shown high expression both in leaf and xylem while Ptr-miR7816, Ptr-miR399, and Ptr-miR7826 are selectively expressed in various vegetative tissues like leaf area (Kuang et al., 2018; Wang R. et al., 2021). One unanticipated finding was that we found some unique miRNAs (Ptr-miR2820, miR2825, Ptr-miR2829, Ptr-miR2836, Ptr-miR2838, and Ptr-miR2840) that have not been reported in previous poplar studies. Our findings will pave the way for more studies into the biological roles of Ptr-miRNAs and the target sites in poplar.

### 4.6 Expression profiles and likely role of PtrCOBLs in abiotic stress and wood formation

The COBL gene family is speculated to have a role in both the plant’s stress response and its ability to produce wood tissues (Yang et al., 2021). High expression levels of these genes have been seen in secondary growth, tension wood, and abiotic stressors, as bolstered by transcriptome datasets. Five different plant organs (**Figure 7**) and 27 different tissue types (**Figure 10**) have different PtrCOBLs expression patterns, as shown by the expression profiling (Sundell et al., 2015; Sundell et al., 2017). In continuation of the trailing results, Cobra-*Like* genes (PtrCOBL2, 3, 10, and 11) registered a positive expression response under cold, heat, drought, and salinity stresses (**Figure 9**). Similar results were reported in *Arabidopsis* (Ko et al., 2006), wheat (Zaheer et al., 2021), *S. spontaneum* (Qiu et al., 2023), and rice (Sun et al., 2022). PtrCOBL2, 3, 6, and 10 and PtrCOBL11 flaunted high representation in stem (node, internode) regions. Studies have shown that stems with increased mechanical strength were more resistant to lodging and disease, which also indicates more biomass production. COBL2 is involved in cell differentiation and is mandatory for cellulose deposition in *Arabidopsis* (Ben-Tov et al., 2018; Niu et al., 2018); accordingly, the PtrCOBL6 and 10 in the AtCOBL2 subgroup showed higher expression abiotic stress response and in wood formation process. Contrary to the expectations, PtrCOBL1, 7, and 9, belonging to the same group, showed the lowest expression, implying an unequal evolution event among the orthologous groups (Yang et al., 2021). PtrCOBL2 and the *BC1* gene, the orthologs of AtCOBL4 found in poplar, rice, and sorghum, control the cellulose content of the secondary cell wall. AtCOBL10 and 11 are involved in pollen tube cell elongation, so showed the highest expression in abiotic stress and cell expansion (Niu et al., 2018; Qiu et al., 2023). Members of the COB group, *BC1* in rice and *BC2* in maize, and *Bna9*, 35, and 41 in rapeseed, were found to be involved in cell wall biosynthesis rendering stem-breaking resistance (Li et al., 2003; Li et al., 2018; Yang et al., 2021). Similar results were found in the current study: PtrCOBL2, 3, 8, 10, 11, and 12 exhibited a higher protein abundance in wood developing tissues. Overall, the AtCOB group was found to be highly involved in cell wall deposition and rendering mechanical strength, in accordance with the previously reported studies (Yang et al., 2021).

For further exploration in this study, correlation coefficients of the expression data were used to conduct co-expression network analysis (Iqbal et al., 2022). Notably, PtrCOBL2 and PtrCOBL3 were the two genes characterized as hub genes in abiotic stress as well as in wood formation-related PtrCOBLs (**Figures 9B–E**, **10C**). Therefore, PtrCOBL2 and PtrCOBL3 could be considered as critical genes in further studies regarding plant stress and secondary growth in poplar. However, some discrepancies in the expression patterns of paralogous gene pairs were discerned (Sangi et al., 2021), e.g., PtrCOBL6/PtrCOBL9 possess a similar structure and conserved motif but showed different expression profiles. PtrCOBL6 has a higher response in wood forming tissues and abiotic stress, while PtrCOBL9 is totally absent in abiotic stress and has lower response in wood forming tissues. Interestingly, PtrCOBL6 has high expression in stems whereas PtrCOBL9 has high expression in roots. As a result, we hypothesize that PtrCOBL genes went through duplication events that may have caused gene mutations, which in turn alter gene function and expression patterns. This phenomenon has also been observed in the ERF family, NAC family, and XTH gene family (Liu et al., 2015; Cheng et al., 2021).

The quantitative polymerase chain reaction (RT-qPCR) was employed to examine the expression of PtrCOBL genes in poplar across a range of tissue types to determine their potential role in controlling cell expansion and abiotic stress. The expression of most genes was consistent, except for PtrCOBL6, PtrCOBL9, and PtrCOBL12 wherein better expressions were found in roots, stems, and YLs, respectively, in comparison to microarray data. This discrepancy could be the result of differences in their experimental settings. Gene ontology (GO) enrichment has bolstered this study (**Figure 6B**). The presumed functions of PtrCOBLs have been fortified with following terms: cellulose microfibril organization, anchored component of membrane, plant-type cell wall organization or biogenesis, cell growth, plant-type secondary cell wall biogenesis, anchored component of plasma membrane, polysaccharide binding, carbohydrate binding, external encapsulating structure organization, extracellular matrix organization, response to abiotic stimulus, and growth. It has been demonstrated that the PtrCOBL genes are predominantly implicated in mechanisms relating to cell distension, biological growth, stress response, and wood development (Li et al., 2018; Zaheer et al., 2021). The findings of the current study suggest that the PtrCOBL genes will be useful in the future development of better abiotic stress-resistant woody plants. Nevertheless, additional research is needed to be done on the functional dissection and potential of COBL genes.

## Data availability statement

The original contributions presented in the study are included in the article/**Supplementary Material**. Further inquiries can be directed to the corresponding authors.

## Author contributions

MS conceived the idea, conducted the analyses, investigation, and wrote the original draft. AA provided the methodology and software. MR and MY contributed the plant materials and to data analysis. QH conducted the literature survey and visualization. MS and M-ZL undertook manuscript review and editing. M-ZL performed supervision and funding acquisition. All authors contributed to the article and approved the submitted version.
