# Supplementary material for: Recent genome resequencing paraded COBRA-Like gene family roles in abiotic stress and wood formation in Poplar
Source: Front Plant Sci. 2023 Sep 15;14:1242836. doi: 10.3389/fpls.2023.1242836 (PMC10540467; doi:10.3389/fpls.2023.1242836)

**Recent Genome resequencing paraded COBL gene family roles in abiotic stress and wood formation in Poplar.**

**Supplementary Figure 1.** Chromosomal and gene locations of the identified PtrCOBL genes.


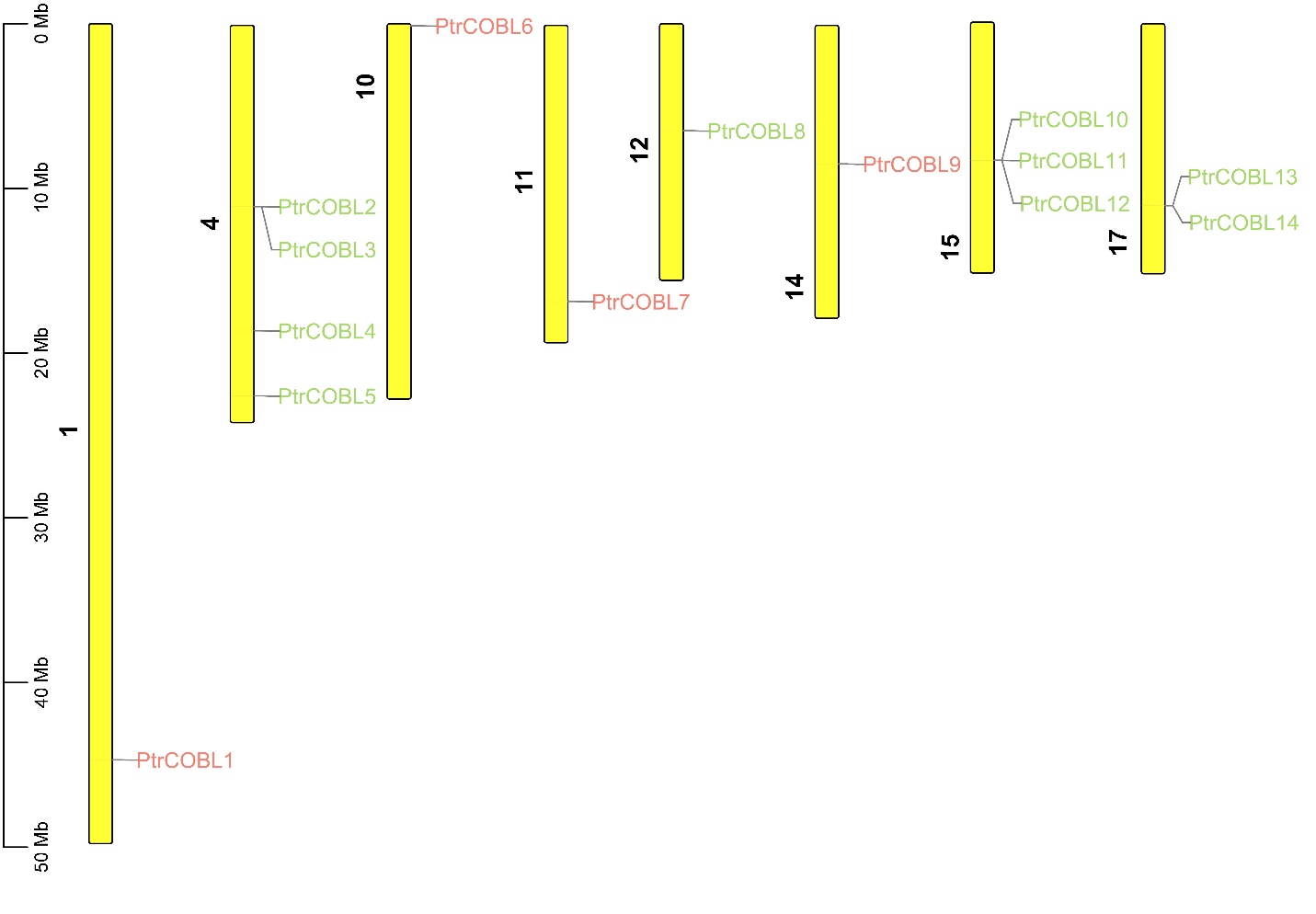


**Supplementary Figure 2.** Global Ptr-miRNA identification and localizations targeting PtrCOBL genes.


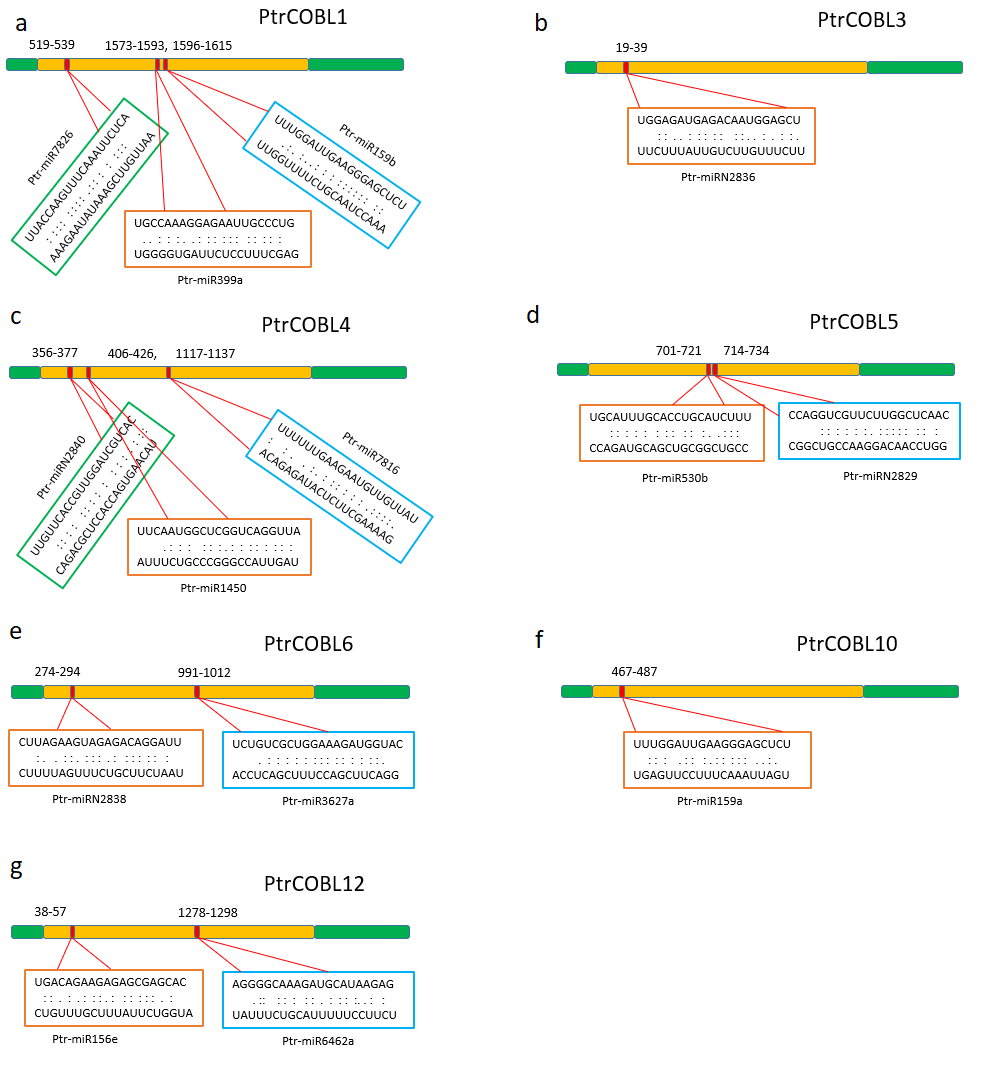

Supplement: Supplementary file 1 [file DataSheet_1.docx]
